# Can Narrative Skills Improve in Autism Spectrum Disorder? A Preliminary Study with Verbally Fluent Adolescents Receiving the Cognitive Pragmatic Treatment

**DOI:** 10.1007/s10936-023-09945-4

**Published:** 2023-05-08

**Authors:** Dize Hilviu, Federico Frau, Francesca M. Bosco, Andrea Marini, Ilaria Gabbatore

**Affiliations:** 1 Department of Psychology, GIPSI Research Group, University of Turin, Via Verdi, 10, 10124 Turin, Italy; 2 Laboratory of Neurolinguistics and Experimental Pragmatics (NEP), University School for Advanced Studies IUSS, Pavia, Italy; 3 Neuroscience Institute of Turin, University of Turin, 10043 Turin, Italy; 4 Department of Languages, Literatures, Communication, Education and Society, Cognitive neuroscience lab, University of Udine, Via Margreth, 3, 33100 Udine, Italy; 5 Claudiana-Landesfachhochschule Für Gesundheitsberufe, 39100 Bolzano, Italy; 6 Research Unit of Logopedics, Faculty of Humanities, University of Oulu, 90014 Oulu, Finland

**Keywords:** Autism spectrum disorder, Verbally fluent ASD, Adolescence, Narrative skills, Pragmatic training

## Abstract

Autism spectrum disorder (ASD) is a neurodevelopmental condition affecting social and communicative skills, including narrative ability, namely the description of real-life or fictive accounts of temporally and causally related events. With this study, we aimed to determine whether a communicative-pragmatic training, i.e., the version for adolescents of the Cognitive-Pragmatic Treatment, is effective in improving the narrative skills of 16 verbally fluent adolescents with ASD. We used a multilevel approach to assess pre- and post-training narrative production skills. Discourse analysis focused on micro- (i.e., mean length of utterance, complete sentences, omissions of morphosyntactic information) and macrolinguistic measures (i.e., cohesion, coherence errors, lexical informativeness). Results revealed a significant improvement in mean length of utterance and complete sentences and a decrease in cohesion errors. No significant change was found in the other narrative measures investigated. Our findings suggest that a pragmatically oriented training may be useful in improving grammatical efficiency in narrative production.

## Introduction

Autism spectrum disorder (ASD) is a complex neurodevelopmental condition that manifests with deficits in social interaction and communicative abilities (DSM-5; American Psychiatric Association, 2013). Impairments are observable in the communicative-pragmatic domain (Angeleri et al., 2016; Baixauli-Fortea et al., 2019; Martzoukou et al., 2017; for a review, see Volden, 2017), which is the ability to use language (Levinson, 1983) and other expressive means such as gestures, facial expressions (Bara, 2010) in a given context appropriately. Pragmatic ability allows interlocutors to go beyond the literal meaning of utterances and to understand their communicative intentions (Grice, 1975, 1991).

Narrative skills are part of pragmatic competence and consist in the ability to describe real-life or fictive accounts of temporally and causally related events (Boudreau, 2008). Narrative skills represent a fundamental aspect for the development of personal identity from late childhood to adolescence (Reese et al., 2010; Steiner & Pillemer, 2018) and the quality of narratives is considered an important predictor of teenagers’ educational achievement (Jones et al., 2018; Spencer et al., 2017) and psychological and mental well-being (Manczak et al., 2014; Reese et al., 2017; Shiner et al., 2021). Furthermore, alterations in narrative development are likely to influence psychosocial outcome from childhood to adolescence (Aguilar et al., 2020). From a linguistic point of view, a narrative requires a combination of micro- and macrolinguistic processes (Marini & Carlomagno, 2004; Pistono et al., 2017; Pritchard et al., 2017). Microlinguistic processes allow for the generation of the narrative at the lexical and the sentence level. Macrolinguistic processes ensure the pragmatic functionality of discourse (i.e., between-sentence processing) through the use of cohesive devices and the generation of coherent episodes within a discourse (Marini & Carlomagno, 2004; Marini et al., 2011).

Previous studies have reported lower levels of narrative abilities in verbally fluent children with ASD (Carlsson et al., 2020; for a meta-analysis, see Baixauli et al., 2016). For instance, at the microlinguistic level, children with ASD produce more grammatical errors and less complete sentences, whereas, at the macrolinguistic level, they may fail to use linking devices correctly (e.g., referential pronouns and conjunctions) to organise story content (Makinen et al., 2014; Westerveld & Roberts, 2017).

Many studies that have assessed narrative production abilities in a cohort of both children and adolescents (up to 18 years old) with ASD reported that they make considerably more micro- (e.g., reduced syntactic complexity) and macrolinguistic (e.g., irrelevant comments and ambiguous references) errors than their typically developing peers (Capps et al., 2000; Losh & Capps, 2003; Losh & Gordon, 2014; Marini et al., 2020). While such difficulties may decrease with age (Iandolo et al., 2020; Norbury et al., 2014), a meta-analysis by Baixauli et al. (2016) found that the narrative performance of children did not differ from the performance of adolescents (up to 15 years of age) with ASD, suggesting the possibility that narrative difficulties in ASD are likely to remain constant from childhood through adolescence.

Consequently, studies investigating the narrative profile of teenagers with ASD exclusively (from 11 to 18 years old) have reported that the occurrence of syntactic oversimplifications persists during adolescence and that the ability to express temporal and causal connections between events is reduced in adolescents with ASD (King & Palikara, 2018). Difficulty in building textual cohesion and managing the coherence of narrative discourse (Canfield et al., 2016; King & Palikara, 2018) make it harder to understand the stories of adolescents with ASD compared to those produced by typically developing peers (de Marchena & Eigsti, 2016).

The impact of narrative impairment on daily communicative interactions has encouraged the development of training programmes devoted to the improvement of such difficulties. For instance, extensive literature on the efficacy of narrative-based treatments for patients with aphasia (Boyle, 2011; for a review, see Dipper et al., 2020), traumatic brain injury (Cannizzaro & Coelho, 2002; for a review, see Steel et al., 2021), children with language disorders (Gillam et al., 2018; for a review, see Favot et al., 2020) and Williams syndrome (Diez-Itza et al., 2018) exists. Previous studies have described examples of individualized interventions addressing narrative difficulties in children with ASD (Favot et al., 2018; Gillam et al., 2015; Petersen et al., 2014). The focus of such training programmes was to help children improve story structure planning and the use of complex syntax and cohesive links (e.g., temporal and causal conjunctions) to construct coherent stories.

Little attention has been directed to specific interventions on narrative abilities in adolescents with ASD. General communicative training programmes for adolescents with ASD use various strategies of intervention, including peer-mediated conversational programmes (Axe, 2018; Bambara et al., 2016; Thomas & Bambara, 2020), social skills group training (Choque Olsson et al., 2017; Dekker et al., 2019; Matthews et al., 2020), and training focused on communicative-pragmatic abilities (Gabbatore et al., 2021). To the best of our knowledge, only one study to date has described training-related improvement of narrative production in adolescents and young adults with ASD (McCabe et al., 2017). The participants received a parental-mediated intervention to enhance the production of their personal narratives of everyday situations (e.g., visiting the hospital, getting lost, etc.). For their study, McCabe and colleagues trained the participants’ parents and provided them with specific recommendations to promote narrative production in daily communicative interactions with their children (see also Peterson et al., 1999 for a similar procedure). Samples of narrative discourse produced by the participants before and after the programme were assessed using a quantitative (e.g., assessment of story grammar complexity, topic maintenance, and occurrence of off-topic comments) and a qualitative approach (i.e., the quality of their stories as perceived by their parents). Overall, an improvement in the adolescents’ narrative skills was observed. The outcome was positive, yet the training did not directly involve the participants with ASD but only their caregivers.

### Aims of the Study

Considering the importance of narrative development for the psychosocial outcome of teenagers, the aim of this study is to determine whether a communicative-pragmatic training programme, i.e., the Cognitive Pragmatic Treatment (CPT; Gabbatore et al., 2015) adapted for adolescents (A-CPT; Gabbatore et al., 2021), is effective in enhancing the narrative skills of a cohort of verbally fluent adolescents with ASD. The aim of the A-CPT programme is to improve communicative-pragmatic skills (e.g., conversational abilities, social awareness, production and comprehension of literal and non-literal communicative acts, such as indirect speech acts and irony) via a variety of expressive means (linguistic, extra-linguistic, e.g., gestures, and paralinguistic, e.g., prosody). The original version of CPT has proven effective in increasing the narrative ability of persons with traumatic brain injury (Parola et al., 2019). Recently, A-CPT has been shown effective in improving pragmatic abilities in a cohort of verbally fluent adolescents with ASD by a pre- post- specific evaluation with the equivalent forms of the Assessment Battery for Communication (Bosco et al., 2012)—in both comprehension and production (Gabbatore et al., 2021). That said, data on the potential positive effect of A-CPT on the narrative skills of individuals with ASD are still missing.

To fill this gap, we assessed the narrative skills of a cohort of adolescents with ASD pre- and post-training using a multilevel approach to discourse analysis (Marini et al., 2011). This approach was effective in capturing discourse errors in children and adults with different profiles of communicative impairment (Marini et al., 2007, 2008, 2010, 2020). We hypothesized that a substantial improvement in narrative skills at the micro- and the macrolinguistic levels would be noted in the study sample after participation in the A-CPT programme.

In addition to the narratives, we assessed cognitive functioning. A recent narrative review (Matthews et al., 2018) found that, in typical and atypical development, the relationship between pragmatics—including narrative skills—and other cognitive variables is not fully clear yet. For example, Kuijper et al. (2015) found that working memory and inhibition were predictive of appropriate referent reintroduction in narrative production tasks; Ketelaars et al. (2012) showed that executive functions were strongly associated with narrative productivity, when controlling for language ability, in children with pragmatic language impairment, while no specific link between such capacities was detected in typically developing children. Analogously, Blom and Boerma (2016) measured narrative comprehension and production in children with Developmental Language Disorder and found an association between working memory and narrative comprehension and production, while in typically developing children working memory was associated with comprehension only. Similarly, Duinmeijer and coauthors (2012) measured narrative variables in two different narrative conditions (i.e., story generation and story retelling) and cognitive skills in a group of children with developmental language disorder compared to a group of typically developing children, and found specific correlations between features of each narrative condition and cognitive abilities. Overall, while the current literature seems to suggest that a number of cognitive functions correlate with (some aspects of) pragmatic skills, including narrative ability (Cannizzaro & Coelho, 2013; Nayar et al., 2018), pragmatics still appears to address specific aspects and is not merely the sum of different cognitive abilities (see also Bambini et al., 2016; Bosco et al., 2019; Bosco et al., 2018c; Bosco & Gabbatore, 2017a; Gabbatore et al., 2017; Parola et al., 2018). This theoretical view is also supported by empirical studies showing that, for example, a training programme specifically designed to address the improvement of cognitive skills (i.e., attention) does not lead to an improvement in terms of pragmatic communication (Youse & Coelho, 2009). Conversely, pragmatic training programmes aimed at improving communication do not cause an amelioration of executive functioning (Gabbatore et al., 2021; Parola & Bosco, 2018) thus indicating that a clear underlying distinction between such processes exists and that they do not simply overlap. Given the complex, and not yet completely clear relationship between these variables, we also assessed a pool of cognitive functions before and after training in order to verify that the effect of the training was specific for the target variable of the study, i.e., narrative ability.

Cognitive assessment was performed using a selection of tasks of the Neuropsychological Evaluation Battery (BVN 12-18; Gugliotta et al., 2009). These tasks assess memory, attention, and executive functions (i.e., *shifting*, *inhibition, updating*). As the above-mentioned cognitive abilities are not a target of A-CPT, the aim of this assessment was to determine whether potential improvement could be observed after specific training for pragmatic ability, which was the programme target variable. Since we used the cognitive assessment as a control measure, we did not expect any specific improvement.

Finally, given the results of previous studies (Duinmeijer et al., 2012; Ketelaars et al., 2012; Kuijper et al., 2015; Matthews et al., 2018), we expect a relationship between narrative variables and cognitive abilities at baseline. However, since we expect an improvement in narrative but not cognitive skills after-training, we suppose these correlations might not persist at T1.

## Material and Methods

### Participants

Eighteen (17 males and 1 female) adolescents with a diagnosis of autism spectrum disorder made by an expert psychiatrist and based on DSM-IV criteria (ASD; American Psychiatric Association, 1994), were enrolled in the study through the collaboration between the research group and two rehabilitation centers in Piedmont (Italy) area, namely Gruppo Asperger Onlus (Turin) and Centro di Riabilitazione Ferrero (Alba). One participant did not complete the training because of personal commitments. Another participant was excluded from analysis due to a serious form of stuttering that made it extremely difficult to understand the recordings and encode the narratives. The final sample included 16 participants (15 males and 1 female; age range 12–18 years, mean 13.94 ± 1.98) with 6 to 13 years of schooling (mean 8.75 years ± 2.02). All participants were native Italian speakers. They were initially enrolled based on their IQ (cut off ≥ 80) as reported in their clinical records. Nevertheless, they were retested with the Italian version of Raven’s Standard Progressive Matrices (Raven, 1938) with reference to the standardized norms for adolescents (Picone et al., 2017). The mean IQ for the sample was 94.56 ± 14.30. Sample characteristics are summarized in Table 1. Individuals were excluded if they were attending an Applied Behavior Analysis rehabilitation programme or other programmes targeting communicative abilities at the time of the present study. Inclusion criteria were adequate linguistic abilities, which were further assessed with the Token test of the Neuropsychological Evaluation Battery (BVN 12-18; Gugliotta et al., 2009), a subtest for linguistic comprehension.

**Table 1:** Demographic characteristics of the sample included in the training program

Demographic variables	
No. of participants	16
Gender	
Males	15
Females	1
Age (years)	
Mean (SD)	13.94 (1.98)
Min–Max	12–18
Education (years)	
Mean (SD)	8.75 (2.02)
Min–Max	6–13
IQ	
Mean (SD)	94.56 (14.30)
Min–Max	80–123

The participants’ families agreed to take part in the training programme after they attended a presentation of the research project held at the rehabilitation center and involving research group members, professionals working at the center, as well as the adolescents and their families. Prior to data collection, the participants and their caregivers gave written, informed consent to participate in the training programme and permit videorecording of the sessions. The participants and their families were provided detailed information about the nature and aims of the study in compliance with the ethical code of the Italian Association for Psychology (AIP) and in accordance with the tenets of the Declaration of Helsinki. The participants and their families were also informed that participation was voluntary, that they could refuse to participate and withdraw from the study at any time, and that data confidentiality would be assured according to current data protection norms and legislation. The project was approved by the Bio-Ethical Committee of the University of Turin, protocol n. 134703.

The training was provided in small groups of 4 to 5 participants, each undergoing the same number of sessions and the same type of training activities. The average attendance rate was 94.27%. The trainers were graduate students on a master’s programme in psychology; they had received training in the structure and the procedures of the A-CPT programme, as well as in the administration of assessment tools for evaluating narrative and cognitive abilities. Assessment and rehabilitation were supervised by a team of experts in pragmatic and cognitive impairment, psychologists, and members of the research team who had developed the original CPT programme.

### A Cognitive Pragmatic Training Programme for Adolescents

We used the version adapted for adolescents (A-CPT; Gabbatore et al., 2021) of the Cognitive Pragmatic Treatment (CPT; Gabbatore et al., 2015), a manualized programme retrievable at https://www.dippsicologia.unito.it/do/gruppi.pl/Show?_id=za8x that has proven effective in enhancing communicative-pragmatic skills in a cohort of verbally fluent adolescents with ASD, aged 12-18. A-CPT is a group training programme theoretically grounded on cognitive pragmatics, a theory on the cognitive and inferential processes underlying human communication. It focuses on communicative-pragmatic abilities, and specifically on a range of skills that allow individuals to communicate efficiently and effectively: linguistic, extralinguistic, and paralinguistic abilities; social appropriateness; awareness; conversational and narrative skills; social and planning abilities. A-CPT includes activities designed to improve participants’ communicative efficiency in both comprehension and production (Table 2). Each session has its focus on a specific aspect of communication and provides participants with an ecological setting where they can practice their pragmatic abilities while simulating daily routine activities. The aim of A-CPT activities is to help participants improve their inferential skills, i.e., their ability to fill the gap between literal and intended meanings (e.g., irony and figurative language). Another aim is to improve the ability to maintain attention through the efficient use of expressive modalities, i.e., language, gestures, facial expressions, prosody, tone, and rhythm of the voice. Such an ability can better emphasize intended meaning and facilitate the understanding of pragmatic phenomena, e.g., in the use of irony, as an identical literal utterance may convey different meanings depending on situational cues (Bosco et al., 2017).

**Table 2:** Outline of the adolescents adapted version of the Cognitive Pragmatic Treatment (A-CPT)

Training session	Designed activities
1 Introduction and overall communicative ability	Introduction to the aims and structure of the A-CPT programme; setting-up of the working group by a self-introduction of each participant, including the description of any perceived difficulty in daily living communication Overview of the communicative-pragmatic ability, via video clips and role-playing tasks, based on daily living situations and depicting all the communicative expressing means
2 Linguistic ability	Video clips and role-playing based on the linguistic expressive modality
3 Extralinguistic ability	Video clips and role-playing based on the gestural modality
4–5 Paralinguistic ability	Video clips, facial expression recognition, tone of voice tasks, role-playing;
6–7 Social appropriateness	Video clips and role-playing focused on social appropriateness and communicative adequacy in different contexts
8 Conversational ability	Video clips, role-playing and exercises focused on the use of conversational rules (i.e., turn-taking topic management)
9 Phone conversation	Audio clips and role-playing focused on telephone conversational rules (i.e., voice only, no paralinguistic and gestural clues, available in live interactions)
10–11 Social ability	Video clips and role-playing focused on the ability to formulate meta-representations with respect to one’s own and others’ mental states
12 Narrative ability and planning	Picture-description task, aimed at eliciting story-telling by providing an adequate amount and type of information
13–14 Overall communicative ability	Video clips and role-playing focused on the overall pragmatic effectiveness, expressed through all the modalities constituting communicative competence
15 Conclusion, awareness and feedback	Conclusions and feedback about the progresses observed along A-CPT i.e., video recording of the salient moments along the sessions where the improvements could be detected were shown to each participant during the group session

The structure of each session remained constant throughout the training programme regardless of the specific topic of the session:

- *Introduction and overview.* Introduction to the current session content, with particular attention to the connection between the current communicative topic and participants’ daily life episodes.
- *Comprehension activities.* These were mostly video clips illustrating brief communicative interactions created ad hoc for the A-CPT programme. Participants were asked to observe two actors interacting in a specific communication modality presented during the session (i.e., linguistic modality in linguistic sessions or gestural modality in extralinguistic sessions and so forth). At the end of each video clip, the participants were invited to comment on the interactions they observed, to stimulate their comprehension of the communicative situations portrayed during the video. Discussion with other group members served to improve discourse coherence and enhance compensatory communication strategies. Self-monitoring and feedback were provided by the therapist and the other group members during the training sessions to guide and support comprehension.
- *Production activities.* These were chiefly role-play activities (i.e., interactive scenarios of everyday situations), in which the participants held in-pairs communicative interactions to improve and strengthen their ability to use contextual elements. The role-play activities also provided the participants with communication strategies and feedback from the therapist and the other group members within a safe group-training setting. The A-CPT programme included specific activities involving paralinguistic (e.g., recognition and production of facial expression, exercises for modulating the tone of voice), narrative (picture description and famous movie plots), and planning abilities (planning of activities and tasks to be performed within a given amount of time).
- *Conclusion and homework.* Assigned at the end of each session, the homework consisted of tasks for practice at home of the communication strategies that had been illustrated during the training session. The activities provided the participants with an opportunity to practice and improve their communicative skills acquired during the A-CPT sessions.

Overall, A-CPT includes of a total of 15 sessions: one session per week, each lasting approximately 90 min, including a 10-min break (Table 2). The original version of CPT (Gabbatore et al., 2015) consists of two sessions per week (12 weeks) for a total of 24 sessions; each session lasts approximately 90 min and includes a 10-min break. See (Bosco et al., 2016; Bosco et al., 2018b; Gabbatore et al., 2017; Sacco et al., 2016) for a more detailed description of the structure of CPT and the content of training sessions.

The length and the content of the original version of CPT (Gabbatore et al., 2015) was adapted in order to make it more suitable for adolescents.

### Assessment Measures

#### Narrative Assessment

Narrative abilities were assessed at T0 (pre-training) and T1 (post-training), i.e., within one week after the end of the training. Discourse samples were elicited using four picture stimuli: two single-picture scenes entitled the Picnic taken from the Western Aphasia Battery (Kertesz, 1982) and the Cookie Theft by Goodglass and Kaplan (1972), and two picture sequences entitled the Flower Pot by Huber and Gleber (1982) and the Quarrel by Nicholas and Brookshire (1993). Each participant was assessed individually in a quiet room at the rehabilitative center. Administration of the stimuli, transcription of the speech samples, and multilevel discourse analysis were performed following the criteria detailed in Marini et al. (2011). Pictures were administered in the same order to all participants using a laptop with the display facing the participant to prevent memory limitations and referent sharing. The participants had to describe the situation depicted in the pictures without using ambiguous words (e.g., *here*, *there*, etc.) as the task administrators stated they were unfamiliar with the stimuli. The narratives were audio-recorded. The speech samples were later transcribed verbatim by one transcriber, with the inclusion of phonological fillers, pauses, false starts, and extraneous utterances. The transcripts were analyzed by the same coder. The duration (in seconds) of each sample was calculated, as well as the total number of *units* and *words*. The term *units* defines the verbalizations produced by a speaker, including well-formed words, non-words (i.e., neologisms such as *tasper instead of *table*), and phonological paraphasias (e.g., *plower instead of *flower*), false starts (e.g., *There is a d-d-d- dog*), sounds, and syllable repetitions. The term *words* refers to well-formed words produced with the exclusion of neologisms and phonological paraphasias. Each transcription was segmented in utterances. Utterance segmentation was carried out taking into account jointly the presence of clear pauses between utterances (*acoustic criterion*), the presence of a complete semantic unit including a main predicate and its arguments (*semantic criterion*), the presence of a grammatically complete sentence with its subordinate clauses (*grammatical criterion*), and the presence of interrupted words or false starts (*phonological criterion*).

Narrative analysis was performed using a multilevel approach to micro- and macrolinguistic features of narrative production (Marini & Carlomagno, 2004; Marini et al., 2011). This procedure has been used in studies on cohorts of children with various clinical conditions (Marini et al., 2007, 2008, 2010), including school-aged children with ASD (Marini et al., 2020).

The microlinguistic analysis focused on three measures:

- Mean length of utterance (MLU) calculated by dividing the total number of words by the number of utterances. Under the assumption that longer utterances require more words and, in principle, more complex grammatical characteristics, MLU provides indirect information about the grammatical skills of the participants.
- Percentage of omissions of morphosyntactic information calculated by dividing the omissions of content words by the number of utterances and multiplying the result by 100. This percentage provides information about the participants’ ability to adequately use the morphosynctactic information required by words while generating sentences.
- Percentage of complete sentences calculated by dividing the grammatically complete sentences by the number of utterances and multiplying the result by 100. An utterance was considered a complete sentence if it did not contain any omissions or substitution of morphemes or words. Therefore, this percentage allows to directly assess the participants’ ability to generate well-formed sentences.

The macrolinguistic analysis focused on three measures:

- Percentage of cohesive errors calculated by dividing the number of cohesive errors by the number of utterances and multiplying the result by 100. A cohesive error included misuse of cohesive ties, such as connectives (e.g., *The man falls from the tree* / *but he hurts himself*), number and gender agreement between pronouns and nouns (e.g., *I saw John* / *and I told her about you*), and abrupt interruptions in an utterance, i.e., *aposiopesis* (e.g., *The man is…* / *The man falls from the tree*). This percentage provides information about the participants’ ability to adequately link consecutive utterances by means of linguistic connectors.
- Percentage of coherence errors calculated by dividing the number of global coherence errors by the number of utterances and multiplying the result by 100. Errors of global coherence included the production of utterances that were repeated (e.g., *There is a man* / *and…* / *There is a man*), fillers (e.g., *There is a…* / *I don’t remember its name*), tangential (i.e., utterances with derailment in the flow of discourse, e.g., *The man falls from the tree* / *I really like trees*) or conceptually incongruent with the story. The percentage of coherence errors provides information about the speakers’ ability to produce utterances that are adequately related to the main gist of the story and therefore of their discourse organization skills.
- Percentage of lexical informativeness calculated as lexical information units (LIUs) in the narratives, such as content and function words that were not only phonologically well-formed but also grammatically and pragmatically appropriate (Marini & Urgesi, 2012). Words scored as errors of any kind and words embedded in fillers, repeated, incongruent or tangential utterances were excluded from the count of LIUs. The percentage of lexical informativeness was calculated by dividing the number of LIUs by the number of words and dividing the result by 100. The percentage of lexical informativeness provides direct evidence of the participants’ ability to convey relevant pieces of information with their words.

Transcriptions were performed by one coder while another coder transcribed 9 randomly-selected stories allowing for the calculation of the degree of riability. The two coders reached almost perfect agreement based on the number of units (Average ICC = 0.996; *p* < 0.001) and utterances (Average ICC = 0.946; *p* < 0.001). Scoring was performed by the coder who had transcribed all the speech samples. A random sample of nine narratives was selected and given to a another coder for calculating interrater reliability. Overall, the two raters reached excellent agreement on percentages of Complete Sentences (Average ICC = 1.00; *p* < 0.001), Coherence errors (Average ICC = 1.00; *p* < 0.001), and on MLU (Average ICC = 1.00; *p* < 0.001). They also reached an almost perfect agreement on the percentage of cohesive errors (Average ICC = 0.925; *p* < 0.001) as well as on the percentages of morphosyntactic information (Average ICC = 0.979; *p* < 0.001) and lexical informativeness (Average ICC = 0.998; *p* < 0.001).

#### Assessment of Cognitive Skills

Cognitive skills were assessed in one session of approximately one hour: the first assessment was conducted a few days before training began (T0); the second session was carried out immediately after training had finished (T1). The cognitive profile was determined using a selection of tasks of the Neuropsychological Evaluation Battery, standardized in Italian for adolescents (BVN 12-18; Gugliotta et al., 2009), as described in Table 3.

**Table 3:** Brief description of the neuropsychological tasks administered pre- and post-training

*Token test, 36 items De Renzi and Faglioni* (1978)
The task assesses linguistic comprehension. The examiner reads a list of instructions of increasing difficulty regarding tokens differing in shape (squares and circles), size (large and small) and color (green, white, yellow, red and blue). The first five sets of instructions are based on the verb ‘touch’, e.g., ‘Touch a circle’ or ‘Touch the red circle’. The last set of instruction increases in difficulty and includes a wider variety of actions, e.g., ‘Before touching the yellow circle, pick up the red square’. Each instruction executed in a correct way is attributed a score of 1, while any instruction executed incorrectly is attributed a score of 0. The total score corresponds to the sum of the score obtained at each item (ranging from 0 to 36)
*Naming task Brizzolara et al.* (1993)
The task assesses the ability to name items. The examiner shows, one by one, 88 black and white pictures, and asks the examinee to name them as fast as possible. The pictures present well-known objects belonging to the following semantic categories: animals, toys, tools, vegetables, cloths, fruits, pieces of furniture, means of transport, music instruments, domestic appliances, professions. The pictures’ name may have high or low degree of iteration frequency in daily communicative interaction (e.g., chair vs. accordion), determining a certain variability in terms of complexity. Each pic correctly named is attributed a score of 1 while when a mistake occurs a score of 0 is attributed. The total score corresponds to the sum of the score obtained at each item (ranging from 0 to 88)
*Digit Span and Corsi block-tapping test Bisiacchi et al.* (2005)
The tasks assess, respectively, verbal and spatial working memory. Specifically, they measure the ability to keep in mind a limited amount of information (numbers and locations/spatial relations between objects, respectively), in a readily available state, for a short period of time. In the *Digit span*, the participant is asked to repeat, after the examiner, sequences of numbers of increasing length. The total score is based on the longest series of numbers for which 2 or more sequences are correctly repeated. Score ranges from 0 to 9. In the *Corsi block-tapping test,* the examinee is presented with 9 wooden blocks arranged irregularly. The examiner taps the blocks in randomized sequences of increasing length (from 2 to 7), and the examinee is required to repeat the same sequence immediately after him/her. Each block-tapping series has three sequences of the same length. The total score is based on the length of the sequence of at least two taps (out of three) that the examinee repeats correctly. The score ranges from 0 to 7
*Immediate and Deferred Recall test for long-term verbal memory task Spinnler and Tognoni* (1987)
The tasks assess the ability to extract and memorize information and recall it, immediately after its presentation and after a short time has elapsed. The examinee is required to repeat the content of a short text after listening to the examiner reading it out loud. The task is repeated once immediately after the examiner has read the text and again about 10 min later (in this time range the examinee is engaged in non-verbal tasks to rule out any possible interference with the present task). The content of the text is organized in main events (i.e., what has happened) and their secondary features (when and where), with different degree of relevance, which correspond to different scores. The total score is separate for immediate and deferred task and in both cases ranges from 0 to 8
*Selective attention Bisiacchi et al.* (2005)
The task assesses the ability to focus on a single or a few items in a given perceptual field, for a certain amount of time. The task material is made of a pattern of geometric shapes (i.e., squares with a line in different angles) displayed on a paper sheet. The examinee is shown the target square on the upper part of the sheet and, after a brief training, is required to mark all the squares on the paper sheet that look exactly like the target one. Time limit is one minute. A score of 1 is given for each square correctly identified and the total score corresponds to the sum (range 0 to 21)
*Tower of London Shallice* (1982)
The task assesses planning ability. It requires the examinee to create a mental representation of the pattern of a set of given items and establish which actions are needed to switch from the baseline to the given goal configuration. The task is administered using a board with pegs and colored wooden balls. The examinee is required, starting from an initial given configuration, to arrange 3 colored balls on three upright sticks according to a series of given patterns pictured on a paper sheet. The instruction says to try to achieve the goal arrangement in as few moves as possible and by following simple given rules (e.g., do not move more than a ring at a time). A score of 1 is attributed each time the examinee sets the balls on the pegs according to the configuration given, within the maximum time of 1 min and without breaking any of the rules. The total score corresponds to the sum of the scores attributed for each configuration (range 0–12)
*Modified card sorting test Nelson* (1976)
The task assesses shifting and inhibitory control and consists of 4 stimulus and 48 response cards displaying several symbols, different in color (red, green, yellow, blue), number (1, 2, 3, 4), and type (triangle, star, cross and circle) of shape. The examinee is requested to sort the response cards so to place each of them below one of the stimulus cards. Each response card has only one feature in common with three of the stimulus cards, and none with the fourth one. The examinees are not given information about the sorting criterion to be used (i.e., shape, color or number), but they are guided to discover the sorting rule at each move. A score of 1 is attributed for each criterion correctly identified and applied 6 times in a row. The total score represents the total number of categories correctly identified (range 0–8)

### Data Analysis

First, a Principal Component Analysis (PCA) with a direct oblimin rotation was conducted in order to explore the structure of the six narrative variables at T0 (pre-training) and T1 (post-training). Afterwards, participants’ performance differences between T0 and T1 were assessed by comparing the narrative scores and cognitive measures with a series of Wilcoxon tests for paired samples. Bonferroni’s correction was used to adjust p-value threshold for multiple comparisons. Spearman’s correlations were performed on measures obtained at T0 and T1 to test for associations among the set of narrative variables and then between narrative and cognitive variables. Bonferroni’s correction was used to adjust p-value threshold for multiple correlations.

## Results

### Principal Component Analysis

The PCA was computed on the six narrative measures at T0 and T1 with a direct oblimin rotation.

Concerning pre-training, the Kaiser–Meyer–Olkin measure verified the sampling adequacy for the analysis (KMO = 0.513). Bartlett’s test of sphericity indicated that correlations between variables were sufficiently large for PCA (χ^2^ = 67.68, *p* < 0.001). An initial analysis was conducted to obtain eigenvalues for each component in the data. Two components had eigenvalues over Kaiser’s criterion of 1 and described 80.86% of the variance. The convergence of the scree plot and Kaiser’s criterion suggested that two components had to be retained in the final analysis. The measures that cluster on the same components suggest that component 1 represents the microlinguisic level and component 2 the macrolinguistic level.

Regarding post-training, the Kaiser–Meyer–Olkin measure verified the sampling adequacy for the analysis (KMO = 0.534). Bartlett’s test of sphericity indicated that correlations between variables were adequate for PCA (χ^2^ = 32.81, *p* < 0.005). Eigenvalues for each component in the data were obtained. Two components had eigenvalues over Kaiser’s criterion of 1 and explained 68.31% of the variance. The convergence of the scree plot and Kaiser’s criterion suggested to retain two components in the final analysis. The measures that cluster on the same components suggest that component 1 represents the microlinguisic level and component 2 the macrolinguistic level. The summary of both structure and pattern matrices at T0 and T1 are reported in Table 4 (as suggested by Graham et al., 2003).

**Table 4:** Principal component analysis was conducted to explore narrative data structure at T0 (pre-training) and T1 (post-training)

	T0**—**Pre-training				T1**—**Post-training			
	Pattern matrix^a^		Structure matrix		Pattern matrix^b^		Structure matrix	
	Component		Component		Component		Component	
	1	2	1	2	1	2	1	2
MLU	.858	.194	.868	.235	− .632	.074	− .625	.013
Omissions of morphosyntactic information	− .907	− .071	− .911	− .114	.893	− .044	.889	.041
Complete sentences	.875	.054	.878	.096	− .828	.020	− .826	− .059
Cohesive errors	− .666	.432	− .645	.400	.814	.200	.833	.278
Coherence errors	.323	.890	.365	.905	− .040	.831	.040	.827
LIUs	.108	− .962	.062	− .957	− .041	− .895	− .127	− .898

Extraction method: Principal component analysis. Rotation method: Oblimin with Kaiser normalization

^a^Convergence for rotation performed in 5 iterations

^b^Convergence for rotation performed in 3 iterations

*MLU* Mean length of utterance; *LIUs* Lexical Information Units

### Narrative Assessment

The Wilcoxon tests for paired samples showed a significant improvement on two microlinguistic measures between pre- and post-training. The narratives contained longer MLU and a higher percentage of complete sentences. Improvement on macrolinguistic measures was noted on the reduced occurrence of cohesive errors. It is worth noticing that omissions of morphosyntactic information, despite not being significantly improved, showed a large effect size. However, the percentage of global coherence errors and LIUs was not significantly different between pre- and post-training (Table 5).

**Table 5:** Performance pre- and post- training at the narrative assessment

Linguistic level	T0—Pre training		T1—Post training		z	p*	r
	Score range Min–Max	Raw score M (SD)	Score range Min–Max	Raw score M (SD)			
*Micro*							
MLU	4.65–10.49	6.20 (1.39)	5.33–12.93	8.12 (2.17)	2.79	**.005**	.70
Omissions of morphosyntactic information	14.82–56.63	35.43 (13.50)	10.10–39.33	24.39 (7.84)	− 2.53	.011	.63
Complete sentences	25.54–68.21	48.24 (12.51)	41.79–74.33	58.85 (8.24)	2.84	**.004**	.71
*Macro*							
Cohesive errors	5.00–60.89	25.22 (13.55)	.00–37.48	16.47 (10.43)	− 2.79	**.005**	.70
Coherence errors	.00–41.96	13.57 (10.21)	.00–25.22	10.83 (8.24)	− .91	.363	.23
LIUs	67.55–91.48	79.37 (7.80)	69.14–87.64	77.96 (5.42)	− .67	.50	.17

Statistically significant results are indicated in bold

^*^p-value threshold was adjusted for multiple comparisons with Bonferroni correction (*p* < .008)

*z* standardized test statistics; *r* effect size; *MLU* Mean length of utterance; *LIUs* Lexical Information Units

Spearman’s correlations on narrative variables at T0 (pre-training) showed a series of significant associations. MLU correlated with the percentage of omissions of morphosyntactic information, the percentage of complete sentences, and the percentage of cohesive errors. The percentage of omissions of morphosyntactic information correlated with the percentage of complete sentences. Finally, the percentage of coherence errors was significantly associated with LIUs. All other correlations were not statistically significant (see Table 6).

**Table 6:** Spearman’s correlation analysis between narrative abilities on pre- training performance

	MLU	Omissions of morphosyntactic information	Complete sentences	Cohesive errors	Coherence errors	LIUs
MLU	–					
Omissions of morphosyntactic information	r = − .70 ***p* < .002**	–				
Complete sentences	r = .69 ***p* < .003**	r = − .90 ***p* < .001**	–			
Cohesive errors	r = − .73 ***p* < .001**	r = .39 *p* = .14	r = − .50 *p* = .05	–		
Coherence errors	r = − .04 *p* = .89	r = − .10 *p* = .71	r = .06 *p* = .83	r = − .02 *p* = .95	–	
LIUs	r = .17 *p* = .53	r = .08 *p* = .78	r = − .05 *p* = .85	r = − .14 *p* = .61	r = − .94 ***p* < .001**	–

Statistically significant results are indicated in bold

*p-value threshold was adjusted for multiple comparisons with Bonferroni correction (*p* < .003)

Degrees of freedom for each correlation = 14

*MLU* Mean length of utterance; *LIUs* Lexical Information Units

Spearman’s correlations between narrative variables at T1 (post-training) revealed only one significant association between the percentage of omissions of morphosyntactic information and the percentage of cohesive errors. All others associations were not statistically significant although a moderate, albeit not significant, correlation was observed between the percentage of omissions of morphosyntactic information and the percentage of complete sentences. See Table 7.

**Table 7:** Spearman’s correlation analysis between narrative abilities on post-training performance

	MLU	Omissions of morphosyntactic information	Complete sentences	Cohesive errors	Coherence errors	LIUs
MLU	–					
Omissions of morphosyntactic information	r = − .37 *p* = .015	–				
Complete sentences	r = .47 *p* = .07	r = − .56 *p* = .02	–			
Cohesive errors	r = − .42 *p* = .11	r = .74 ***p* < .001**	r = − .46 *p* = .08	–		
Coherence errors	r = − .12 *p* = .66	r = − .15 *p* = .58	r = − .16 *p* = .54	r = − .05 *p* = .86	–	
LIUs	r = − .12 *p* = .65	r = − .12 *p* = .65	r = − .14 *p* = .61	r = − .35 *p* = .18	r = − .50 *p* = .05	–

Statistically significant result is indicated in bold

*p-value threshold was adjusted for multiple comparisons with Bonferroni correction (*p* < .003)

Degrees of freedom for each correlation = 14

*MLU* Mean length of utterance; *LIUs* Lexical Information Units

### Cognitive Assessment

We compared the performance scores on the cognitive tasks administered pre- and post-training to determine whether differences could be found. As expected, there was no significant improvement in cognitive skills between pre- and post- training (Table 8).

**Table 8:** Performance (raw scores) pre- and post- training at the Neuropsychological Evaluation Battery (Italian standardization for pre-adolescents and adolescents, BVN 12-18)

Task	T0—Pre training		T1—Post training		z	p*	r
	Score range Min–Max	Raw score M (SD)	Score range Min–Max	Raw score M (SD)			
*BVN 12–18*							
Token test	16–36	30.25 (5.42)	27–36	31.31 (3.46)	.88	.34	.22
Naming	55–78	68.06 (6.79)	60–84	72.19 (7.52)	2.57	.01	.64
Digit span	3–8	4.56 (1.21)	4–7	4.62 (.96)	.26	.79	.07
Corsi block-tapping	4–6	5.25 (.77)	4–7	5.44 (1.03)	.76	.49	.19
Long term memory–immediate & delayed recall	0–8	5.64 (2.52)	3–8	6.25 (2.02)	.11	.91	.03
Selective cancellation task	1–20	11.31 (5.87)	2–20	14.25 (5.00)	2.42	.015	.61
Tower of London	4–12	9.12 (2.19)	6–12	10.19 (1.72)	1.91	.06	.48
Modified card sorting test	1–8	5.31 (2.33)	1–8	6.12 (2.30)	1.44	.15	.36

*p-value threshold was adjusted for multiple comparisons with Bonferroni correction (*p* < .006)

*z* standardized test statistics; *r* effect size

### Correlation Analysis Between Narrative and Cognitive Abilities

Spearman’s correlation analyses at T0 (pre-training) are summarized in Table 9. The performance scores at the Naming task correlated with the omissions of morphosyntactic information.

**Table 9:** Spearman’s correlation analysis on pre- training performance of narrative and cognitive abilities

	MLU	Omissions of morphosyntactic information	Complete sentences	Cohesive errors	Coherence errors	LIUs
Token test	r = .44 *p* = .09	r = − .50 *p* = .05	r = .64 *p* = .008	r = − .14 *p* = .61	r = − .27 *p* = .32	r = .26 *p* = .33
Naming	r = .53 *p* = .03	r = − .75 ***p*** < **.001**	r = .69 *p* < .005	r = − .09 *p* = .75	r = .26 *p* = .33	r = − .25 *p* = .35
Digit Span	r = .02 *p* = .94	r = − .26 *p* = .33	r = .15 *p* = .58	r = .02 *p* = .94	r = .15 *p* = .59	r = − .14 *p* = .61
Corsi block-tapping	r = .06 *p* = .83	r = .10 *p* = .70	r = − .25 *p* = .36	r = .05 *p* = .84	r = − .58 *p* = .02	r = .43 *p* = .10
LTM	r = .60 *p* = .01	r = .48 *p* = .06	r = .65 *p* = .007	r = − .37 *p* = .16	r = − .13 *p* = .64	r = .11 *p* = .69
Selective cancellation task	r = − .02 *p* = .95	r = .04 *p* = .88	r = − .16 *p* = .56	r = .20 *p* = .45	r = − .41 *p* = .12	r = .27 *p* = .32
Tower of London	r = .20 *p* = .45	r = − .45 *p* = .08	r = .42 *p* = .10	r = − .09 *p* = .74	r = − .37 *p* = .16	r = .36 *p* = .17
MCST	r = .52 *p* = .04	r = − .17 *p* = .54	r = .21 *p* = .43	r = − .35 *p* = .18	r = − .68 *p* < .004	r = .71 *p* < .002

Statistically significant result is indicated in bold

*p-value threshold was adjusted for multiple comparisons with Bonferroni correction (*p* < .001)

Degrees of freedom for each correlation = 14

*MLU* Mean Length of Utterances; *LIUs* Lexical Information Units; *LTM* Long term memory—immediate & delayed recall; *MCST* Modified Card Sorting Test

No significant correlation was observed at T1 (post-training), see Table 10.

**Table 10:** Spearman’s correlation analysis on post- training performance of narrative and cognitive abilities

	MLU	Omissions of morphosyntactic information	Complete sentences	Cohesive errors	Coherence errors	LIUs
Token test	r = .40 *p* = .13	r = − .43 *p* = .10	r = .26 *p* = .33	r = − .14 *p* = .61	r = − .09 *p* = .73	r = .03 *p* = .90
Naming	r = − .02 *p* = .93	r = .00 *p* = .99	r = − .25 *p* = .35	r = .22 *p* = .41	r = .18 *p* = .50	r = .17 *p* = .69
Digit Span	r = .17 *p* = .54	r = − .46 *p* = .07	r = .39 *p* = .14	r = − .02 *p* = .94	r = .44 *p* = .09	r = − .43 *p* = .10
Corsi block-tapping	r = .37 *p* = .15	r = − .48 *p* = .06	r = .39 *p* = .13	r = − .12 *p* = .69	r = − .14 *p* = .61	r = .04 *p* = .88
LTM	r = .38 *p* = .14	r = − .01 *p* = .98	r = − .09 *p* = .75	r = .07 *p* = .81	r = − .17 *p* = .53	r = .05 *p* = .85
Selective cancellation task	r = .02 *p* = .93	r = − .16 *p* = .54	r = .18 *p* = .66	r = .29 *p* = .28	r = − .28 *p* = .30	r = − .02 *p* = .94
Tower of London	r = .34 *p* = .19	r = − .09 *p* = .75	r = − .09 *p* = .74	r = − .02 *p* = .95	r = − .11 *p* = .69	r = .19 *p* = .47
MCST	r = .22 *p* = .41	r = − .30 *p* = .25	r = .17 *p* = .52	r = − .25 *p* = .34	r = − .14 *p* = .61	r = − .04 *p* = .90

*p-value threshold was adjusted for multiple comparisons with Bonferroni correction (*p* < .001)

Degrees of freedom for each correlation = 14

*MLU* Mean Length of Utterances; *LIUs* Lexical Information Units; *LTM* Long term memory—immediate & delayed recall; *MCST* Modified Card Sorting Test

## Discussion

With the present study, we investigated whether the narrative ability of a cohort of verbally fluent adolescents with ASD could be improved after the administration of the Cognitive Pragmatic Treatment adapted for adolescents (A-CPT; Gabbatore et al., 2021). A-CPT has recently proved to be successful in increasing communicative pragmatic skills in adolescents with ASD (Gabbatore et al., 2021), as assessed with the equivalent forms of the Assessment Battery for Communication (Angeleri et al., 2012, 2015; Bosco et al., 2012), in which the target variables investigated pertained the comprehension and production of a variety of pragmatic phenomena, conveyed using different expressive means (including use of gestures and paralinguistic cues in addition to language per se). Narrative ability is actually part of pragmatic competence but its focus is mainly on linguistic production, at both micro- and macro-linguistic levels, and the main interest regards the capacity to describe accounts of related events (Boudreau, 2008). Narrative ability is essential for everyday life as it allows to increase social inclusion and promotes professional attainments; therefore, research in this field has a high impact on the personal well-being of individuals with alterations of this ability, e.g., ASD. To the best of our knowledge, however, no data are available regarding the potential effects of such a rehabilitation programme on narrative production skills in adolescents with ASD. To fill this gap, we introduced before (T0) and after (T1) training the assessment of narrative production using a multilevel approach for the analysis of micro- and macrolinguistic aspects of narrative discourse (Marini et al., 2011). The within-group analyses showed a significant post-training improvement on two microlinguistic and one macrolinguistic measure. These results will be discussed in light of previous studies on language development and functioning in persons with ASD.

The analysis of the narratives at the microlinguistic level showed a substantial increase in MLU and complete sentences from T0 (pre-training) to T1 (post-training). This improvement suggests that the greater grammatical efficiency, measured through syntactic accuracy, contributed to enhancing MLU production. It is worth noticing that the omissions of morphosyntactic information approached significance, as observable also by the large effect-size, suggesting that also this variable exhibited a mild improvement with the training. MLU, omissions of morphosyntactic information, and syntactic accuracy were related to each other in the pre-training assessment (T0), indicating that before the programme, longer utterances allowed for more complete sentences with fewer omissions of morphosyntactic information and, conversely, shorter utterances were linked to less complete senteces characterized by more omissions of morphosyntactic information. However, in the post-training assessment (T1), the associations between microlinguistic variables were no longer significant. This could be an effect of the training, which specifically improved specific microlinguisitc variables (i.e., MLU and syntactic accuracy).

Overall, these findings suggest that A-CPT may indirectly improve the grammatical skills of adolescents with ASD. This observation is shared by previous studies (Gillam et al., 2015; Petersen et al., 2014) that found improvements in the use of grammar in children with ASD attending narrative treatment sessions focused on linguistic complexity (e.g., use of subordinating and coordinating conjunctions, adverbs, etc.) and story structure. Finally, a major component of A-CPT involves tasks devoted to strengthening pragmatic abilities expressed by linguistic means, such as conversation and roleplay. Since linguistic production was encouraged during the entire course of the programme, it is not surprising that some features of training, albeit pragmatically oriented, may have led to improvement on microlinguistic measures.

Concerning the macrolinguistic level of discourse processing, we observed a decrease in the percentage of cohesive errors in the post-training narratives. There was a significant negative correlation between this macrolinguistic variable and MLU in the pre-training assessment, suggesting that before receiving the training participants produced more cohesive errors and this affected the production of shorter MLU. Nevertheless, after treatment, the situation was different: cohesive errors were not associated with MLU but were positively correlated with the percentage of omissions of morphosyntactic information. This suggests that cohesive processing was likely influenced by an overall improvement in grammar after training. Concerning the percentage of global coherence errors and the percentage of LIUs, we observed no substantial improvement after training. This lack of improvement in the coherence index was unexpected, especially considering that a previous study reported an improvement in narrative coherence (increased topic maintenance and a reduction in off-topic comments) in adolescents with ASD after a parent-mediated intervention that promoted the production of personal narratives in verbally fluent persons with ASD (McCabe et al., 2017). However, the modality of intervention and the assessment method in McCabe and collegues’ study (2017) differed from ours. For instance, the narrative intervention they described did not directly involve the adolescents with ASD but rather only their parents and caregivers. By contrast, the participants in our study were actively engaged in the training activities of the A-CPT programme. In addition, assessment of narrative discourse was performed using settings, tasks, and coding procedures that cannot be strictly compared between the two studies.

Regarding the percentage of LIUs, the absence of post-training improvement might be explained by the fact that A-CPT addresses a multimodal concept of pragmatics that comprises, in addition to language, other expressive means such as body gestures or tone of the voice. For our study, we focused on narrative ability expressed through language.

The difference in outcomes for cohesion, coherence, and LIUs suggests that these macrolinguistic features are to some degree independent of one another. For example, cohesion appears to be more related to the microlinguistic level, as supported by the association between cohesive errors and omissions of morphosyntactic information and also by the PCA results (for similar findings regarding participants with fluent aphasia, see also Andreetta & Marini, 2015), whereas coherence and LIUs are likely to be more strictly related to the domain of pragmatics. In the pre-training assessment, the higher percentage of LIUs was related to a lower percentage of global coherence errors (as showed by the strong negative correlation). Admittedly, pragmatic ability covers a broad set of skills (Cummings, 2005). While some are directly targeted by the A-CPT programme (e.g., the use of gestures, paralinguistic cues, social appropriateness), pragmatic features more closely related to discourse production are less emphasized during treatment. This might explain why these macrolinguistic features did not improve with treatment. In contrast, since language use was one of the expressive means targeted by the tasks in the A-CPT program, this might have enhanced not only microlinguistic discourse features but also narrative cohesion.

As regards cognitive assessment, in light of the complex and not fully clear interplay of cognitive functions and pragmatics in typical and atypical development (see Hyter, 2017; Matthews et al., 2018), a cognitive battery was administered before and after training to verify that its effect was specific for the target variable of the study (narrative ability) and not for the other variables investigated. As expected, we found no significant difference between T0 and T1 in the assessed cognitive measures. Before training, only the Naming task, which evaluates the ability to name items, was positively associated with omissions of morphosyntactic information. This suggests that the ability to perform an appropriate lexical selection leads to process more adequately morphosyntactic information at the sentence level (Andreetta & Marini, 2015).

This correlation is no longer present after training suggesting that the improvement in narrative measures pertains only to pragmatics and narrative skills as a result of the pragmatic training. Consequently, the fact that participants’ cognitive profile did not improve after receiving A-CPT confirms that this specific set of variables was not affected by the training.

Overall, such results indicate a specific improvement in the target skills addressed by the training programme, namely, communicative-pragmatic ability, rather than a general effect due to mere participation in social activities. Our data support the notion that pragmatic ability, which is also influenced by other cognitive functions, addresses something specific beyond the sum of other cognitive skills (see Bambini et al., 2016; Bosco et al., 2019; Bosco et al., 2018a; Bosco & Gabbatore, 2017a, 2017b).

To our best knowledge, this is the first study to investigate the effect of A-CPT on the narrative abilities of adolescents with ASD, with a focus on micro- and macrolinguistic features of discourse production. Given the lack of studies regarding the effectiveness of training to improve pragmatic and narrative skills in adolescents with ASD, our results—first of all the very high attendance rate of the participants (up to 94.27%)—contribute to filling this gap in this line of research.

The present study has some limitations. First, although cognitive assessment served as a control measure, we did not compare our sample’s performance to a control group of adolescents with ASD who did not undergo A-CPT. Future studies should include a control group to exclude the possibility of generic improvement. Second, normative data regarding narrative analysis for this age group are not available. This leaves open the question whether the baseline performance of our study sample was already so high that it precluded detecting pre-and post-training differences in their narrative performance. In this circumstance, normative data would be highly useful. Third, while the structure of the narrative task minimizes any learning effect, for further studies it would be useful to develop equivalent forms of narrative stimuli to rule out any bias when adopting test–retest procedures. Even though in the present study we did not detect any learning effect as the cognitive performance scores remained overall stable in the two assessment phases, this is a factor that would deserve attention in future studies. The number of assessment tools for which equivalent/parallel forms are available is very limited; nevertheless, being able to control for practice and learning effects at different stages would be beneficial also for the cognitive tasks. In addition, the order of stimuli administration (i.e., the order of the stories) was not randomized. Since the pictures were shown in the same order to all participants, we were unable to control for this effect. Finally, it should be noted that the sample size could have impacted the results. However, the difficulty in recruiting participants with the required strict demographic and clinical characteristics is a known limitation in the field, often resulting in reduced sampe sizes not ensuring sufficient statistical power (Tager-Flusberg, 2004).

Finally, it would be interesting and an intriguing suggestion for further studies, to include—in addition to narrative elicitation—broader communicative measures (e.g., ADOS, Lord et al., 2012; CCC-2, Bishop, 2003) or quality of life indexes, e.g., Short-Form Health Survey (SF-36; Ware Jr & Sherbourne, 1992) or Coping Response Inventory for Youth (CRI- Youth, Moos, 1993), in order to determine the degree to which the improvement obtained due to the participation to A-CPT is generalizable to broader patterns of social-communicative skills.

In conclusion, this is the first study to focus on the improvement of narrative skills in adolescents with ASD after participation in a training programme designed to improve their pragmatic skills. This contributes to filling an important gap in the literature with potential impact on the occupational success, independent living, and community inclusion of persons with ASD, considering the importance of narrative skills for teenagers’ educational achievement and psychosocial outcome. Our findings suggest that narrative difficulties may persist in ASD during adolescence and that a pragmatically oriented training programme such as A-CPT may be useful in improving grammatical and cohesive efficiency in narrative discourse production tasks. The lack of statistically significant improvement in the ability to maintain overall coherence in the narratives and to convey relevant pieces of information through words suggests the need for training programmes that improve the ability of children and adolescents with ASD to adequately plan, monitor, and produce samples of narrative discourse that are perceived as informative and communicatively appropriate by their interlocutors. This will be an area of focus for future research.

## Data Availability

The data are not publicly available for privacy or ethical restrictions.
